# Supplementary material for: Patient-derived organoids and orthotopic xenografts of primary and recurrent gliomas represent relevant patient avatars for precision oncology
Source: Acta Neuropathol. 2020 Oct 3;140(6):919–49. doi: 10.1007/s00401-020-02226-7 (PMC7666297; doi:10.1007/s00401-020-02226-7)
Supplement: Supplementary file 1 — Supplementary material 1 (PDF 5114 kb) [file 401_2020_2226_MOESM1_ESM.pdf]

SUPPLEMENTARY MATERIAL

Patient-derived organoids and orthotopic xenografts of primary and recurrent gliomas represent relevant patient avatars for precision oncology

AUTHORS

Anna Golebiewska[1#] · Ann-Christin Hau[1#] · Anaïs Oudin[1#] · Daniel Stieber[1,2] · Yahaya A. Yabo[1,3] · Virginie Baus[1] · Vanessa Barthelemy[1] · Eliane Klein[1] · Sébastien Bougnaud[1] · Olivier Keunen[1,4] · May Wantz[1] · Alessandro Michelucci[1,5,6] · Virginie Neirinckx[1] · Arnaud Muller[4] · Tony Kaoma[4] · Petr V. Nazarov[4] · Francisco Azuaje[4] · Alfonso De Falco[2,3,7] · Ben Flies[2] · Lorraine Richart[3,7,8,9] · Suresh Poovathingal[6] · Thais Arns[6] · Kamil Grzyb[6] · Andreas Mock[10,11,12,13] · Christel Herold-Mende1[0] · Anne Steino[14,15] · Dennis Brown[14,15] · Patrick May[6] · Hrvoje Miletic[16,17] · Tathiane M. Malta[18] · Houtan Noushmehr[18] · Yong-Jun Kwon[9] · Winnie Jahn[19,20,21] · Barbara Klink[2,9,19,20,21,22] · Georgette Tanner[23] · Lucy F. Stead[23] · Michel Mittelbronn[6,7,8,9] · Alexander Skupin[6] · Frank Hertel[6,24] · Rolf Bjerkvig[1,16] · Simone P. Niclou[1,16,*]

# Equal contribution
*simone.niclou@lih.lu

AFFILIATIONS

[1] NORLUX Neuro-Oncology Laboratory, Department of Oncology, Luxembourg Institute of Health, 84, Val Fleuri, 1526 Luxembourg, Luxembourg
[2] National Center of Genetics, Laboratoire National de Sante, 3555 Dudelange, Luxembourg
[3] Faculty of Science, Technology and Medicine, University of Luxembourg, 4367 Belvaux, Luxembourg
[4] Quantitative Biology Unit, Luxembourg Institute of Health, 1445 Strassen, Luxembourg
[5] Neuro-Immunology Group, Department of Oncology, Luxembourg Institute of Health, 1526 Luxembourg, Luxembourg
[6] Luxembourg Centre for Systems Biomedicine, University of Luxembourg, 4367 Belvaux, Luxembourg
[7] Luxembourg Center of Neuropathology, Luxembourg, Luxembourg
[8] Laboratoire National de Sante, National Center of Pathology, 3555 Dudelange, Luxembourg
[9] Department of Oncology, Luxembourg Institute of Health, 1526 Luxembourg, Luxembourg
[10] Division of Experimental Neurosurgery, Department of Neurosurgery, University of Heidelberg, 69120 Heidelberg, Germany
[11] Department of Medical Oncology, National Center for Tumor Diseases (NCT) Heidelberg, Heidelberg University Hospital, 69120 Heidelberg, Germany
[12] Department of Translational Medical Oncology, National Center for Tumor Diseases (NCT) Heidelberg, German Cancer Research Center (DKFZ) Heidelberg, 69120 Heidelberg, Germany
[13] German Cancer Consortium (DKTK), 69120 Heidelberg, Germany
[14] DelMar Pharmaceuticals, Inc., Vancouver, BC, Canada
[15] DelMar Pharmaceuticals, Inc., Menlo Park, CA, USA
[16] Department of Biomedicine, University of Bergen, 5019 Bergen, Norway
[17] Department of Pathology, Haukeland University Hospital, Bergen, Norway
[18] Department of Neurosurgery, Henry Ford Health System, Detroit, MI 48202, USA
[19] German Cancer Consortium (DKTK), 01307 Dresden, Germany
[20] Core Unit for Molecular Tumor Diagnostics (CMTD), National Center for Tumor Diseases (NCT), 01307 Dresden, Germany
[21] German Cancer Research Center (DKFZ), 69120 Dresden, Germany
[22] Institute for Clinical Genetics, Faculty of Medicine Carl Gustav Carus, Technische Universitat Dresden, Fetscherstrase 74, 01307 Dresden, Germany
[23] Leeds Institute of Medical Research at St James's, St James's University Hospital, Leeds, UK
[24] Department of Neurosurgery, Centre Hospitalier Luxembourg, 1210 Luxembourg, Luxembourg

SUPPLEMENTARY FIGURES

SUPPLEMENTARY FIGURE 1

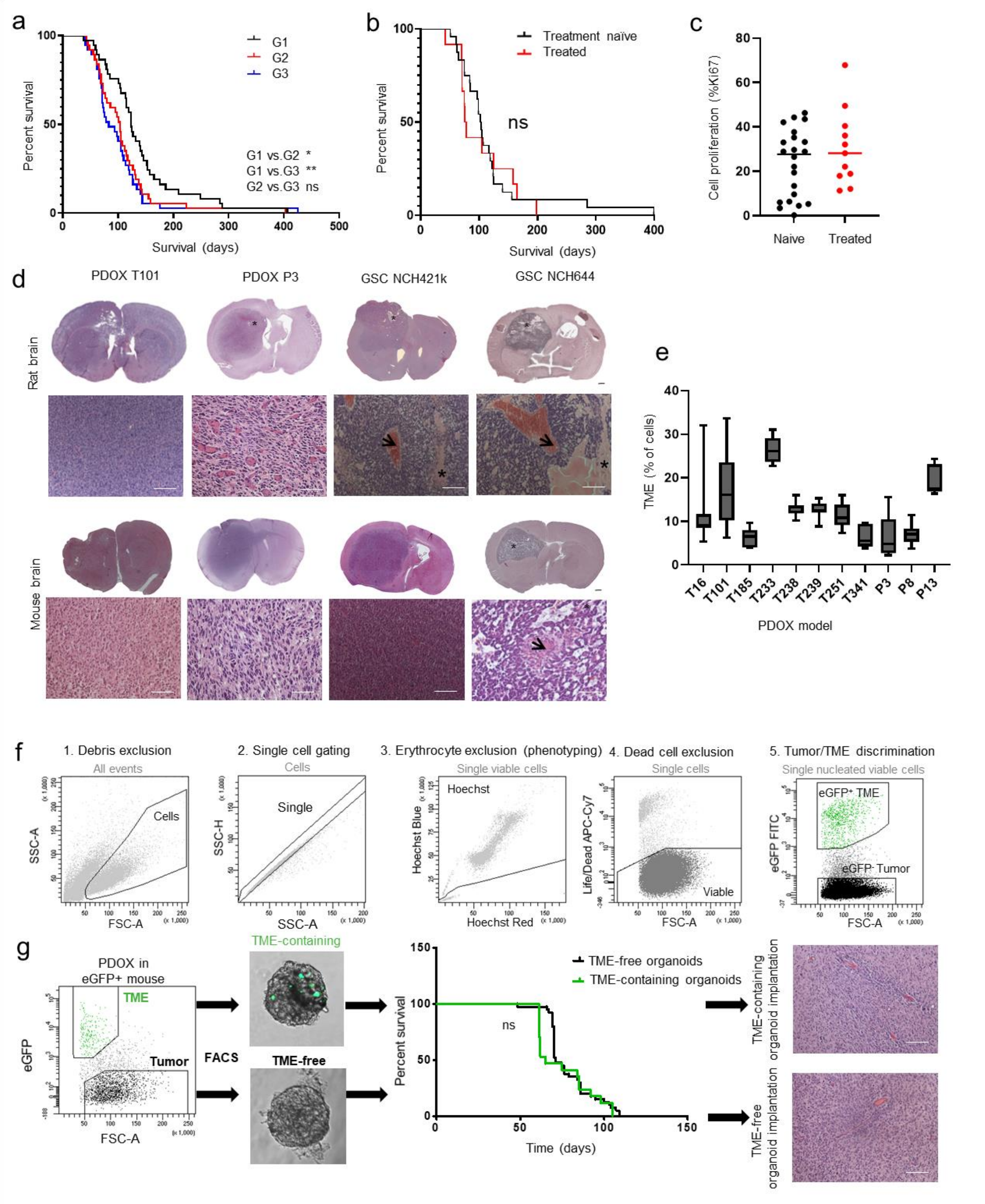

Supplementary Figure 1. Characterization of glioma PDOX models. a Kaplan-Meier survival curves of PDOXs at generation 1 (G1), 2 (G2) and 3 (G3) for those models where G ≥ 3 was reached (n=37). Mean survival of each model per generation was plotted in each group. See **Table S1** for details; (* p-value <0.05, ** p-value <0.01, Wilcoxon signed-rank test). **b** Kaplan-Meier survival curves of PDOXs separated into treatment-naïve and treated gliomas. ns = not significant (Log-rank and Wilcoxon signed-rank tests). **c**

Comparison of cell proliferation in PDOXs (mean %Ki67 per model) derived from treatment-naïve and treated patient tumors. No statistically significant difference was observed (unpaired two-tailed t-test). **d** Additional examples showing variance of histopathological features in rats and mice. Comparison of PDOXs (T101, P3) and GSC line-derived xenografts (NCH421k, NCH644) with strong invasive (T101), towards intermediate (P3, NCH421k) and angiogenic (NCH644) features (arrows = microvascular proliferation, stars = pseudopalisading necrosis, black bar = 1 mm, white bar = 100 µm). **e** Quantification of mouse derived tumor microenvironment (TME) content in the tumor core of PDOX models. Mouse cells were recognized by flow cytometry as hCD90 negative viable cells. **f** Gating strategy for flow cytometry**.** Example is shown for PDOX P3 in $eGFP^{+}$ NOD/SCID mice (1) Cells were distinguished from debris based on the Forward Scatter (FSC) and Side Scatter (SSC). (2) Cell aggregates were gated out based on their properties displayed on the SSC area (SSC-A) versus height (SSC-H) dot plot. (3) For multicolor phenotyping, erythrocytes were excluded on the 'Hoechst Red'/'Hoechst Blue' dot plot in the linear scale. Hoechst staining was omitted for sorting due to increased toxicity (4) Dead cells were recognized by their strong positivity for the dead cell marker (5) In PDOXs, human tumor cells were recognized as eGFP negative, compared to the eGFP positive mouse non-malignant cells (tumor microenvironment = TME). **g** TME-free organoids were derived from FACS-sorted PDOX T16 eGFP- tumor cells only. As control, TME-containing organoids were generated from simultaneous FACS of eGFP- tumor and eGFP+ TME cells. Kaplan-Meier survival curves represent survival of mice across 5 generations per group (G1-G5, total n = 40 for TME-containing and n =12 for TME-free passaging). No statistical difference was observed (Log-rank and Wilcoxon signed-rank tests). Representative histology of PDOXs obtained by TME-containing and TME-free passaging (generation 4) shows no change in tumor phenotype.

SUPPLEMENTARY FIGURE 2

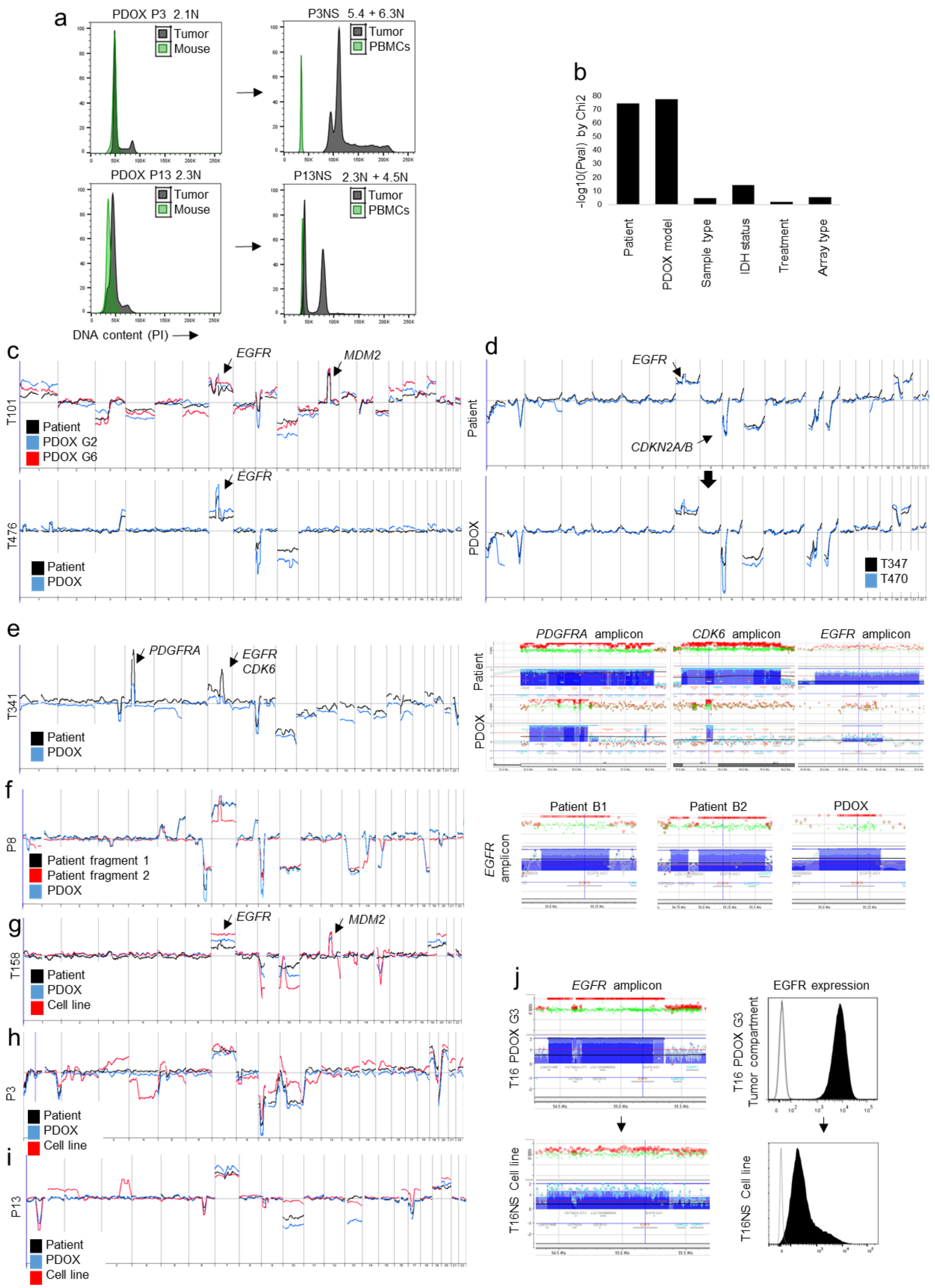

Supplementary Figure 2. Genetic aberrations in glioma preclinical models. a Ploidy analysis of tumor cells in P3 and P13 glioma PDOXs and PDOX-derived GSC cultures (P3NS, P13NS). Mouse cells were used as diploid control (2N) in PDOX samples, PBMCs were used as diploid control (2N) for *in vitro* cultures. Examples are shown for cultures in passage < 10. See more examples in **Table S1**. Note strong aneuploidization of P3 and P13 GSC lines upon *in vitro* cultures (P3NS, P13NS). **b** Statistical analysis of array-CGH data. Chi^2 test for independence reveals limited impact of sample type, treatment and array type on genetic profiles. Individual tumor genetic aberrations and *IDH1* mutation status are main sources of variation. **c** Examples of array-CGH profiles of GBM patient tumors and corresponding PDOX models are shown for T101 and T476. Genetic aberrations were recapitulated over serial transplantations. See **Table S2** for detailed description. **d** Array-CGH profiles of longitudinal samples (T347-T470) of GBM patient LIH0347 showing the same genetic aberrations upon recurrence. These profiles are largely recapitulated in PDOX models, only an additional 1p31.1-p11.2 loss was detected in PDOX T470. **e** Array-CGH profiles of GBM T341 patient tumor and corresponding PDOX. PDOX model was derived from an additional *MDM4/CDK6*-amplified clone with different chromosomal breakpoints. Right panels show presence of different amplicons in the patient tumor and corresponding PDOX. **f** Array-CGH profiles of GBM P8 patient tumor fragments and corresponding PDOX. Analysis of 2 tumor fragments revealed intra-tumoral genetic heterogeneity and different *EGFR* amplicon. **g-i** Array-CGH profiles of GBM patient tumors, corresponding PDOXs and *in vitro* GSC lines for T158 (g), P3 (h), P13 (i). Additional aberrations occurred upon *in vitro* passaging. Note that PDOX T158 arose from an additional *MDM2*-amplified, *EGFR*-non amplified clone, not detected in the patient sample. **j** *In vitro* passaging of T16 tumor cells as GSC line (T16NS) led to loss of *EGFR* amplicon (array-CGH, left panel) and decreased EGFR expression (flow cytometry, right panel).

SUPPLEMENTARY FIGURE 3

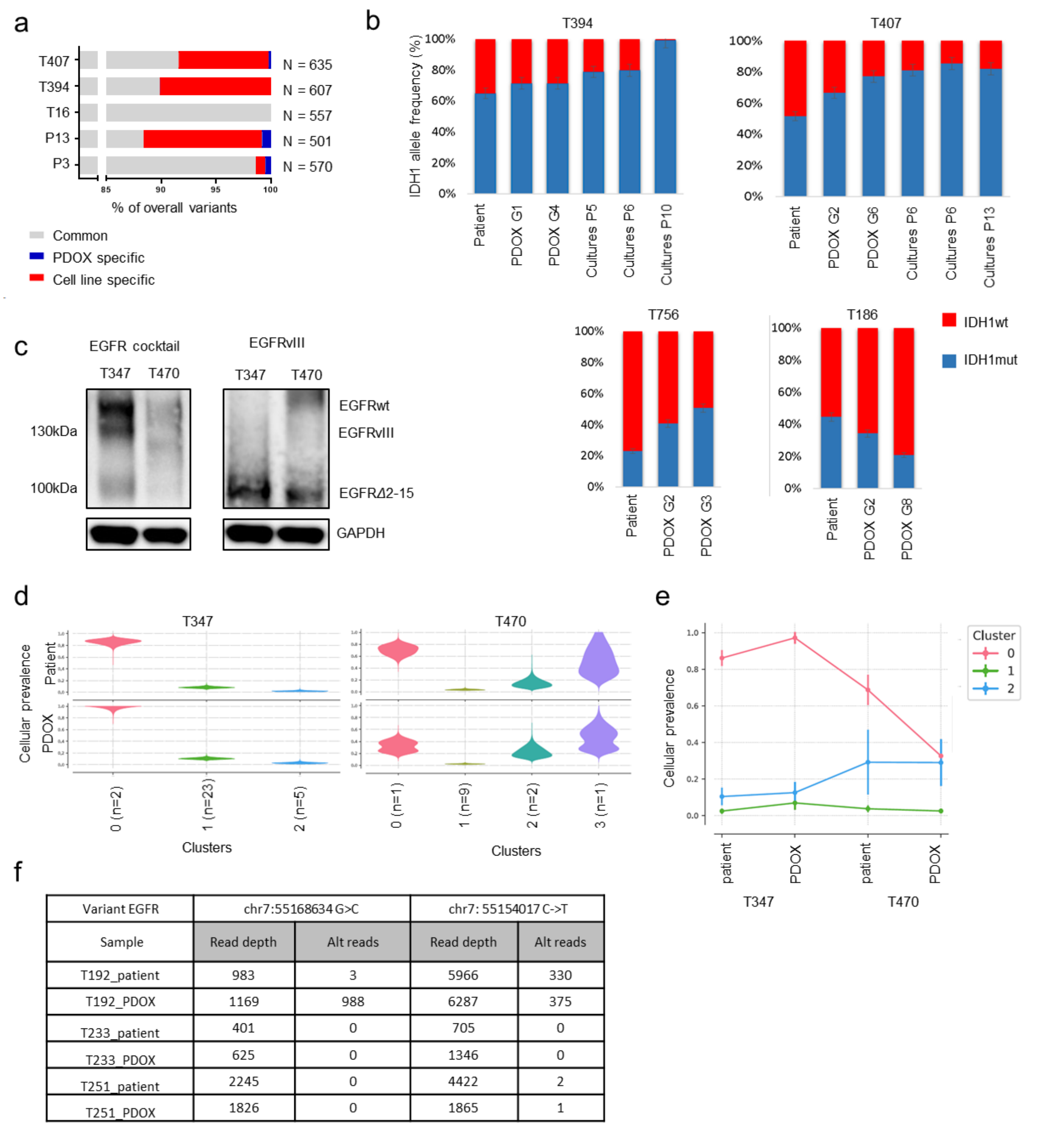

f

Variant EGFR	chr7:55168634 G>C		chr7: 55154017 C->T	
Sample	Read depth	Alt reads	Read depth	Alt reads
T192_patient	983	3	5966	330
T192_PDOX	1169	988	6287	375
T233_patient	401	0	705	0
T233_PDOX	625	0	1346	0
T251_patient	2245	0	4422	2
T251_PDOX	1826	0	1865	1

Supplementary Figure 3. Recapitulation of genetic heterogeneity in glioma PDOXs. a Recapitulation of overall variants in PDOX-derived cell lines detected by targeted sequencing. Cell lines were compared to respective PDOX models. The number of total variants detected for each cell line and PDOXs is displayed. **b** Digital PCR-based analysis of *IDH1 wild-type* and *R132H* fractions in IDH1mut gliomas and corresponding preclinical models. **c** Western blots against EGFR (antibody cocktail recognizing wild-type and structural variants) and EGFRvIII proteins in PDOX-derived organoids T347 and T470 (patient LIH0347) show wildtype EGFR protein as well as the structural variant EGFRΔ2-15, which is recognized by EGFRvIII antibody, despite decreased molecular weight. **d** Cellular prevalence estimates from PyClone representing clonal subpopulations detected in patient tumors and respective PDOXs. Examples shown for longitudinal samples (T347, T470) of patient LIH0347. Each cluster of mutations was computationally inferred to reflect a subclone. Number of genetic variants contributing to each clone is depicted. **e** Cellular prevalence estimates from PyClone representing clonal subpopulations detected in longitudinal samples of patient LIH0347 and the respective PDOXs. Each line represents a cluster of mutations computationally inferred to reflect a subclone. Only genetic variants detected in all samples were considered for analysis. **f** Evolutionary

dynamics of *EGFR* genetic variants. Targeted DNA sequencing revealed longitudinal evolution of *EGFR* genetic variants in LIH0192 patient tumors and PDOXs derived thereof. A specific subclonal variant was present only in T192 patient tumor and was enriched in the respective PDOX (55168634 G->C). Another genetic variant present in T192 patient tumor and PDOXs at the subclonal level was selected out during disease progression (chr7:55154017 C>T). Comparison of overall read depths suggests that these variants are discordantly inherited upon tumor recurrence in patients and PDOX derivation, supposedly via unequal distribution of extrachromosomal DNA.

SUPPLEMENTARY FIGURE 4

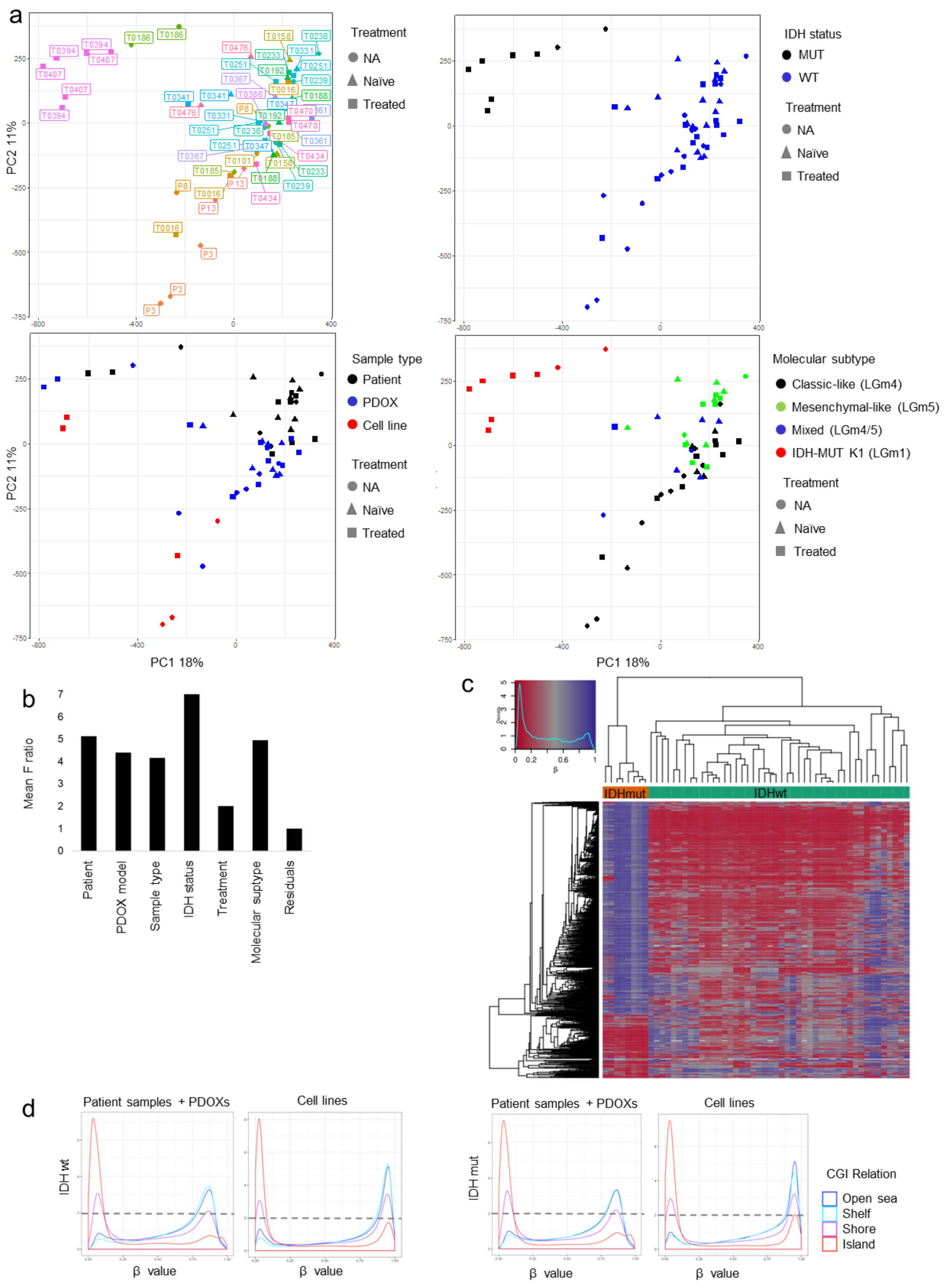

Supplementary Figure 4. Recapitulation of DNA methylation profiles in glioma PDOXs. a Principal component analysis of DNA methylation profiles between glioma patient samples, PDOXs and cell lines derived thereof based on 450k and EPIC arrays (overlapping regions between arrays only). PCA graphs highlighting treatment, IDH status, sample types, and molecular subtypes are displayed. **b** Single factor

ANOVA based on global beta-value distributions reveals IDH mutation status as a main source of variation in the DNA methylation cohort. **c** Heatmap representing 1000 most variable features in DNA methylation shows genomic loci differentially methylated between IDH1mut versus IDHwt patients and preclinical models. **d** beta-value distributions are very similar between IDH1mut and IDHwt tumors (patient samples and PDOX models), in accordance with the G-CIMP low status of the IDH1mut gliomas. IDHwt and IDH1mut GSC lines increase DNA methylation at numerous sites corresponding to open seas, shelfs, and shores.

SUPPLEMENTARY FIGURE 5

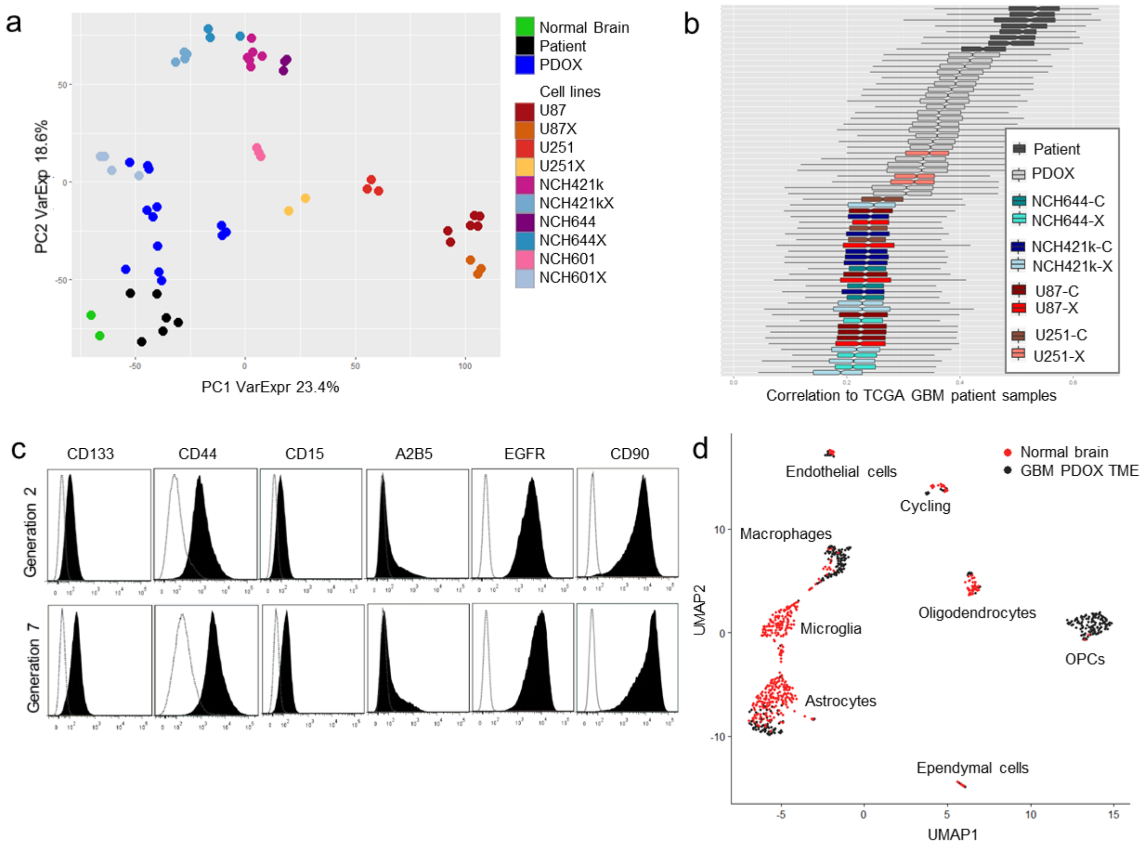

Supplementary Figure 5. Gene expression profiles in glioma preclinical models. a Principal component analysis indicating similarity of genome-wide gene expression profiles between normal human brain, glioma patient samples, PDOXs, GSC lines (NCH421k, NCH644) and classical glioma lines (U87, U251) grown *in vitro* or as corresponding xenograft ('X')**.** Human specific arrays were applied for transcriptome analysis. **b** Correlation of gene expression profiles to TCGA GBM patients shows close resemblance of PDOXs to patient tumors at the transcriptomic level. **c** Stem-cell associated marker expression profiles were interrogated by flow cytometry in tumor cells of PDOXs over serial transplantation. Example shown for PDOX T101. **d** Single cell RNA-Seq of mouse brain showing overall gene expression relationship between cells of normal brain (red) and upon GBM implantation (shown for PDOX P8) (black). Identified TME subpopulations are depicted.

SUPPLEMENTARY FIGURE 6

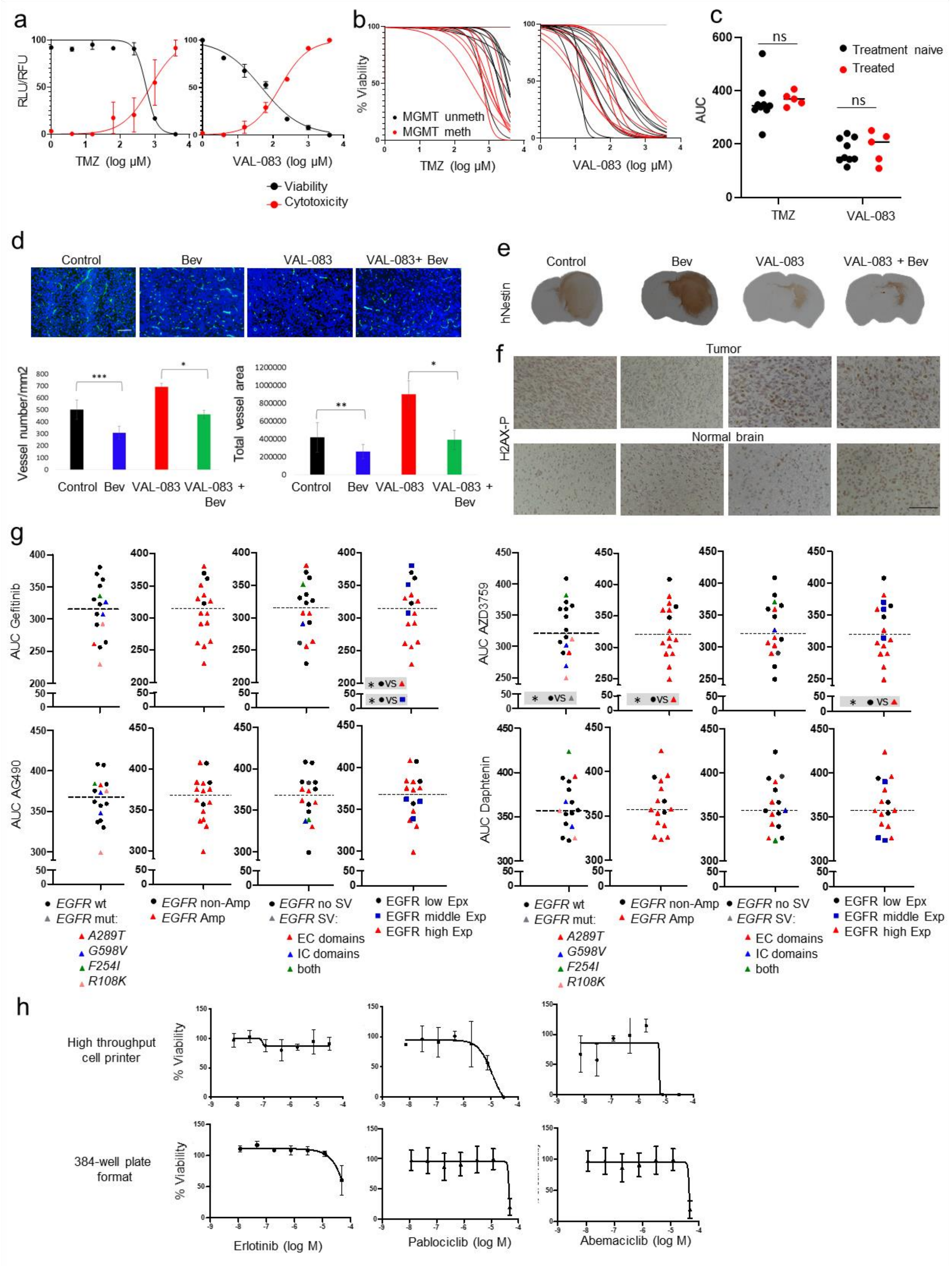

Supplementary Figure 6. Drug testing regimens in organoids and PDOXs *in vivo*. a Quantification of cell viability and toxicity displayed as normalized and log transformed RLU/RFL. Examples are shown for TMZ and VAL-083 response in P3-derived organoids (n = 3). **b** Response curves (non-linear fit, n = 3) of individual PDOXs to TMZ and VAL-083 treatment displayed as %-Viability normalised to untretated control.

MGMT promoter unmethylated and methylated tumors are shown in black and red respectively. **c** Mean AUC upon exposure to TMZ and VAL-083 in PDOX models derived from treatment-naïve and treated tumors. ns = not significant (unpaired t-test). **d** Blood vessels *in vivo* were visualized with mouse specific anti-CD31 staining (in green). Tumor was defined as nuclei dense area (nuclei in blue = DAPI). Representative pictures are shown for each experimental group (Scale bar = 100μm). Quantification of vessel number per mm^2 upon treatment and area covered by vessels confirmed normalization of the tumor vasculature in Bevacizumab (Bev) treated mice (Mean±SD, $*p_{value}<0.05$, $***p_{value}<0.01$, $****p_{value}<0.001$, n = 5-6 for Control and Bev groups, n = 2 for VAL-083 treated groups, 3-5 pictures were taken per each tumor, unpaired t-test). The statistical analysis between Control and VAL-083 treated groups is not shown due to major differences in tumor volumes, leading to lower aberrations in tumor vasculature of the small VAL-083 treated tumors. **e** Representative sections of PDOX stained against human-specific Nestin (n = 3). **f** IHC for H2AX-P in PDOX sections (brown nuclei = H2AX-P). Counterstaining for nuclei with hematoxyline. Induction of H2AX-P was observed in VAL-083 treated tumors. Minor induction of H2AX-P was also observed in a subpopulation of normal brain cells. Scale bar = 100 μm. **g** Quantification of AUC upon exposure to EGFR inhibitors: Gefitinib, AG490, AZD3759 and Daphtenin ($*p_{value} < 0.05$, unpaired t-test); wt = wildtype, mut = mutated, Amp = amplified, SV = structural variant, exp = protein expression. Experiments were performed twice with 3 technical replicates each. *See* **Supplementary Table 8** for mean AUC +/- SEM. **h** Response curves (non-linear fit, n = 2) of PDOX T434 to EGFR and CDK4/6 inhibitors displayed as %-Viability +/- SEM. Similar treatment responses were observed with the two protocols applied.

SUPPLEMENTARY REFERENCES

1 de Souza, C. F. *et al.* A Distinct DNA Methylation Shift in a Subset of Glioma CpG Island Methylator Phenotypes during Tumor Recurrence. *Cell reports* **23**, 637-651, doi:10.1016/j.celrep.2018.03.107 (2018).

1 de Souza, C. F. *et al.* A Distinct DNA Methylation Shift in a Subset of Glioma CpG Island Methylator Phenotypes during Tumor Recurrence. *Cell reports* **23**, 637-651, doi:10.1016/j.celrep.2018.03.107 (2018).

2 Verhaak, R. G. *et al.* Integrated genomic analysis identifies clinically relevant subtypes of glioblastoma characterized by abnormalities in PDGFRA, IDH1, EGFR, and NF1. *Cancer cell* **17**, 98-110, doi:10.1016/j.ccr.2009.12.020 (2010).

3 Wang, Q. *et al.* Tumor Evolution of Glioma-Intrinsic Gene Expression Subtypes Associates with Immunological Changes in the Microenvironment. *Cancer cell* **32**, 42-56 e46, doi:10.1016/j.ccell.2017.06.003 (2017).

SUPPLEMENTARY TABLES

Supplementary Table 1. **Clinical data corresponding to patients of which PDOX models were derived.** Clinically relevant patient information is displayed in columnt C-J, such as age at tumor collection, sex, tumor localization based on MRI, treatment information, histological and molecular diagnosis (https://www.molecularneuropathology.org/mnp) of patient tumors. IDH status as assessed by NGS and MGMT promoter methylation status from Illumina Infinium HumanMethylation BeadChips.
Further, PDOX model relevant info is presented in column K to O, such as implanted tissue type, generations reached, stable survival time, cell proliferation index and ploidy.
If available, PDOX-derived GSC lines are specified in columns P-Q; NA – Not available.

PDOX model	Patient	Age at collection	Sex	Tumor localization	Treatment received prior PDOX	Histological diagnosis	Molecular diagnosis	IDH1 status (patient)	MGMT methylation (patient)	Implantation	PDOX generation reached	PDOX survival time (days)	Proliferation index in PDOX (%Ki67 +/- SD)	Ploidy PDOX	PDOX-derived cell line	Ploidy cell line
P3	BER0003	64	Male	NA	NA	GBM Grade IV	NA	WT	methylated	spheroids	>G10	42.5 +/- 3.6	54.7 +/- 3.7	2.1N	P3NS	5.4N + 6.3N
P8	BER0008	64	Female	NA	NA	GBM Grade IV	GBM, IDHwt, RTK I	WT	methylated	spheroids	>G10	57.4 +/- 5	38.3 +/- 10.8	2.2N		
P13	BER0013	NA	Female	NA	NA	GBM Grade IV	NA	WT	methylated	spheroids	>G10	36.5 +/- 4	42.9 +/- 10.8	2.3N	P13NS	2.3N + 4.5N
T16	LIH0016	52	Female	Fronto-parietal right	TMZ	GBM Grade IV	GBM, IDHwt, mesenchymal	WT	unmethylated	spheroids	>G10	75 +/- 6.5	18.9 +/- 5.8	(2.1N) + 3.4N	T16NS	3.8N
T101	LIH0101	60	Male	NA	NA	GBM Grade IV	NA	WT	methylated	spheroids	G7	101 +/- 11	13.4 +/- 4.8	(2N) + 4.6N		
T158	LIH0158	70	Female	Frontal right	no prior treatment	GBM Grade IV	GBM, IDHwt, mesenchymal	WT	methylated	spheroids	G5	77+/- 18	35.2+/-3	2.3N	T158NS	2.4N
T185	LIH0185	76	Female	NA	no prior treatment	GBM Grade IV	GBM, IDHwt, RTK II	WT	methylated	spheroids	G6	89 +/- 10	19.4 +/- 6.9	2.2N		
T186	LIH0186	34	Male	NA	no prior treatment	Anaplastic Oligodendroglioma Grade III	Glioma, IDHm, high grade astrocytoma	Mutant R132H	methylated	fresh spheroids/tissue	G9	159 +/- 43	33+/-4.8	2.1-2.4N		
T188	LIH0188	68	Male	NA	no prior treatment	GBM Grade IV	GBM, IDHwt, RTK II	WT	unmethylated	spheroids	G5	64 +/- 10	37.1+/- 2.9	2.3N		
T192	LIH0192	42	Female	Frontal left	no prior treatment	GBM Grade IV	GBM, IDHwt, mesenchymal	WT	unmethylated	spheroids	G6	66 +/- 6	26.5 +/-8	2.4N		
T226	LIH0188	69	Male	NA	radiotherapy +TMZ	GBM Grade IV	NA,low tumor content	WT	NA	spheroids	G3	142 +/- 23	NA	NA	T226NS	2.0N + 3.7N
T233	LIH0192	42	Female	Frontal left	radiotherapy + TMZ	GBM Grade IV	GBM, IDHwt, mesenchymal	WT	unmethylated	spheroids	G4	121 +/- 9	11.3	2.3N		
T238	LIH0238	42	Male	NA	no prior treatment	GBM Grade IV	GBM, IDHwt, inflamatory tissue	WT	methylated	spheroids	G3	119 +/- 32	22.1	(2.1N) + 3.7N		
T239	LIH0239	80	Male	NA	no prior treatment	GBM Grade IV	GBM, IDHwt, mesenchymal	WT	methylated	spheroids	G3	124 +/- 25	4.5+/-4.9	2.3N		
T251	LIH0192	43	Female	Frontal left	radiotherapy +TMZ	GBM Grade IV	GBM, IDHwt, RTK II /mesenchymal	WT	unmethylated	spheroids	G6	72 +/- 11	36.1 +/- 7.7	2.3N		
T281	LIH0281	51	Female	Parietal Left	no prior treatment	GBM Grade IV	GBM, IDHwt, mesenchymall/RTKII	WT	methylated	spheroids	G3	117 +/- 13	5.3 +/-0.41	2.0N + 3.5N		
T304	LIH0304	53	Male	Frontal left	no prior treatment	GBM Grade IV	GBM, IDHwt, RTK II /I	WT	unmethylated	spheroids	G3	84 +/- 15	6.01	2.2N + 3.2N +4.4N		
T331	LIH0331	84	Male	Fronto-temporo-parietal left	no prior treatment	GBM Grade IV	GBM, IDHwt, mesenchymal	WT	methylated	spheroids	G4	116 +/- 5	33.2 +/- 2.73	2.0N		
T341	LIH0337	75	Female	Temporal right	no prior treatment	GBM Grade IV	GBM, IDHwt, RTK I	WT	methylated	spheroids	G4	50 +/- 5	37.7 +/- 6.1	2.1N + 3.65N		
T347	LIH0347	41	Male	Frontal left	no prior treatment	GBM Grade IV	GBM, IDHwt, RTK II	WT	unmethylated	spheroids	G3	119 +/- 21	42.1+/-1.9	2.2N		
T356	LIH0281	52	Female	Parietal Left, para-sagittal	radiotherapy +TMZ	GBM Grade IV	GBM, IDHwt, mesenchymal	WT	methylated (low tumor content)	spheroids	G4	162 +/- 45	28.17 +/- 4.49	2.1N		
T361	LIH0361	69	Female	Temporal left, para-brainstem	no prior treatment	GBM Grade IV	GBM, IDHwt, RTK II	WT	methylated	spheroids	G5	75.5 +/- 8	46.3+/-1.6	2.3N		
T363	LIH0363	84	Female	Occipital lobe right	no prior treatment	GBM Grade IV	NA	WT	NA	spheroids	G3	138 +/- 2	6.36	2.1N		
T367	LIH0367	69	Male	Temporal left	no prior treatment	GBM Grade IV	GBM, IDHwt, RTK II	WT	methylated	spheroids	G2	140 +/- 0	29.7+/-3.1	2.3N		
T384	LIH0384	50	Female	Temporo-Occipital left	no prior treatment	GBM Grade IV	GBM, IDHwt, mesenchymal	WT	methylated	spheroids	G2	225 +/- 51	NA	NA	T384NS	3.65N
T386	LIH0386	51	Male	Frontal left	no prior treatment	GBM Grade IV	GBM, IDHwt, RTK I	WT	unmethylated	spheroids	G4	116 +/- 21	28.9+/-0.72	2.1N		
T394	LIH0394	45	Female	Fronto-basal right	radiotherapy	GBM Grade IV	Glioma, IDHm, high grade astrocytoma	Mutant R132H	methylated	fresh spheroids/tissue	G5	70 +/- 9	67.8+/-8.3	2.1-2.3N + 3.5N	T394NS	3.1N
T407	LIH0394	45	Female	Fronto-basal right	radiotherapy + Avastin	GBM Grade IV	Glioma, IDHm, high grade astrocytoma	Mutant R132H	methylated	fresh spheroids/tissue	G4	68 +/- 4	49.5+/-6.8	2.1N + 3.9N	T407NS	3.1N +3.5N
T434	LIH0304	54	Male	Frontal left	radiotherapy + TMZ	GBM Grade IV	GBM, IDHwt, RTK II	WT	unmethylated	spheroids	G6	44 +/- 3	40.4 +/- 11.43	2.0N + 2.3N +3.8N		
T470	LIH0347	42	Male	Frontal left	radiotherapy + TMZ	GBM Grade IV	GBM, IDHwt, RTK II	WT	unmethylated	spheroids	G4	72 +/- 8	17.98 +/- 0.88	2.2N		
T476	LIH0476	75	Male	Fronto-parietal left	no prior treatment	GBM Grade IV	GBM, IDHwt, RTK II/I	WT	unmethylated	spheroids	G4	63 +/- 6	44.3+/-3.8	2.3N		
T515	LIH0515	46	Male	Frontal-right	no prior treatment	Anaplastic Oligodendroglioma Grade III	NA	Mutant R132H	NA	fresh spheroids/tissue	G3	413 +/- 16	NA	NA		
T591	LIH0384	52	Female	Temporo-Occipital right	radiotherapy +TMZ + Avastin	GBM Grade IV	GBM, IDHwt, RTK II /mesenchymal	WT	unmethylated	spheroids	G3	108 +/- 3	22.08+/-12.72	2.2N + 3.5N		
T744	LIH0744	69	Male	Bifrontal	no prior treatment	GBM Grade IV	NA	WT	NA	spheroids	G3	103 +/- 4	28.9+/-23.5	2.2N		
T756	LIH0756	46	Male	Multifocal left	radiotherapy	GBM Grade IV	Glioma, IDHm, high grade astrocytoma	Mutant R132H	methylated	fresh spheroids	G5	73 +/- 14	12.09 +/-1.62	2.2N + 2.9N + 3.9N		
T772	LIH0615	54	Female	Fronto-temporal left	radiotherapy + TMZ	GBM Grade IV	GBM, IDHwt, RTK II	WT	methylated	spheroids	G4	106 +/- 6	32.1+/-1.2	2.1N		
T784	LIH0784	56	Male	Frontal left	no prior treatment	GBM Grade IV	GBM, IDHwt, RTK I	WT	methylated	spheroids	G4	136 +/- 16	43.5+/-3.3	2.1N		
T797	LIH0797	55	Female	Left occipital	no prior treatment	GBM Grade IV	GBM, IDHwt, RTK I	WT	unmethylated	spheroids	G3	126 +/- 9	9.65 +/-0.40	2.2N + 3.9N		
T831	LIH0831	51	Female	Bifrontal	no prior treatment	GBM Grade IV	GBM, IDHwt, RTK II	WT	methylated	spheroids	G3	75 +/- 7	3.46 +/- 2.33	NA		
T832	LIH0831	51	Female	Bifrontal	no prior treatment	GBM Grade IV	GBM, IDHwt, RTK II	WT	methylated	spheroids	G2	123 +/- 2	0.25 +/- 0.04	2.1N		

Supplementary Table 2 **List of antibodies used in the study.**

*Flow cytometry test 106 cells/100µl

Epitope	Conjugate	Species reactivity	Clone	Supplier	Concentration used/test*
CD31	-	mouse	390	Millipore	IHC:1/200
Nestin	-	human	10C2	Millipore	IHC:1/200
Vimentin	-	mouse/rat/human	EPR3776	Epitomics	IHC:1/200
Ki67	-	mouse/rat/human	SP6	ThermoScientific	IHC: 1/100
H2AX-P	-	mouse/rat/human	9718T	Cell Signaling	IHC: 1/500
Anti-mouse -IgG	HRP	mouse		GE Healthcare LNA931V/AG	WB: 1/10 000
Goat anti-rat IgG	Alexa Fluor 555	rat		Invitrogen	IHC:1/1000
Anti-mouse IgG	HRP	mouse		Dakocytomation	Kit concentration
Anti-rabbit IgG	HRP	rabbit		Dakocytomation	Kit concentration
Anti-Mouse IgG	Biotinylated	horse		Vestor labs, BA-2000	IHC:0.2ug/ml
CD15/SSEA-1	Alexa Fluor 647	human	MC-480	Biolegend	5µl/test
CD31	Dy590 (PE-TR)	human	MEM-05	Immunotools	10µl/test
CD44	PE-Cy7	human/mouse	IM7	eBioscience	1.2µl/test
CD45	PE-Cy7	human	HI30	Immunotools	5µl/test
CD90	PECy7/APC	human	5.00E+10	BD Bioscience	5µl/test
CD133	PE /APC	human	293C3/AC 133	Miltenyi	10µl/test
A2B5	APC/PE	human/mouse	105-HB29	Miltenyi	10µl/test
EGFR	PE	human	EGFR.1	BD Bioscience	20µl/test
Lamin A/C	PE	human	sc-7292	Santa Cruz	20µl/test
EGFR	-	human	ocktail R19/	ThermoScientific	WB: 1/1000
GAPDH	-	human	D16H11	Cell Signaling	WB: 1/1000

Supplementary Table 3 **Chromosomal aberrations of glioma patient samples and corresponding PDOX models and GSC lines.**

Modifications in genome structure as identified by array-CGH are shown for each patient tumor [++ = amplification (Log2 Ratio>2), + = gain (Log2 Ratio >0.25), - = loss (Log2 Ratio < -0.25), -- = deletion (Log2 Ratio< -1)]. If present, changes of genetic aberrations of patient profiles are shown for PDOX models and cell lines in column c and d respectively . Genetic aberrations determined only from EPIC methylation arrays are highlighted with red background.

PDOX model	Patient tumor	PDOX specific changes vs. patient	Cell line specific changes vs. PDOX
P3	+[Chr7, 19p, 20q], -[1p36-1p34.1, 1q21.1-q44, -5p15.33-31, Chr9, Chr10, 11p15-14, 20p] --CDKN2A/B		+(3q, Chr21, Chr22), - (4q, Chr11)
P8	++EGFR, +[5p15.3-p12, 5q31.1-q35.3(clone1), Chr7, 8q24], -[6q21-q27, Chr10, 13q13.3-q34, 15q21.2-q23(clone1), 18q21.2-q22.1], --CDKN2A/B	clone 1 selection	
P13	+(Chr7, Chr19, Chr20), -(6q16.3-q21, Chr10, 17q11-12), --CDKN2A/B	- (1p21.1-p31.2, Chr13)	+(1q, 2p, 3q, 16q, Chr17), 0 (Chr10, Chr13)
T16	++[EGFR, MDM2(clone2)], +7q, - [6q, Chr10, 12q21.1-q24.3, 13q11-q31.1], --CDKN2A/B	**G1**: selection of additional clone: ++MDM4, 0 [MDM2, 12q21.1-q24.3]	0 EGFR
T101	++[EGFR, MDM2] +[Chr1, Chr7, Chr9, Chr13, Chr17, Chr19] -[3q, Chr4, Chr10, Chr11, 14q11, Chr15], --CDKN2A/B.	**G6**: +Chr16, -Chr6	
T158	++EGFR, +[Chr7, Chr19, Chr20], -Chr10, --CDKN2A/B	**G1:** selection of additional clone : ++MDM2, +[12q15-q21.1], - [10p15.3-p12.31, 10q11.21-q26.3, 15q13.1-q21.3], --CDKN2A/B, 0 EGFR	- (12q24.31-q24.33), 0 (Chr19)
T185	++EGFR, +[Chr7, Chr20] –[10q, Chr22] --CDKN2A/B		
T186	complex genome ++[MYCN, CDK4]		
T188	++EGFR, +[Chr7, Chr19, Chr20] –[1p36.31-p36.13, 6p21.32, Chr10, Chr13] --CDKN2A/B	0 (Chr13)	
T192	++[EGFR, 2q34], +[1q21.2-24.2, Chr7, Chr19, Chr20], - [9p24.3 -13.3, Chr10]		
T226	low tumor content	++EGFR, +(partial Chr5, Chr7), -(partial Chr1, partial Chr6, Chr10, Chr14, partial Chr16, partial Chr19, Chr20), --CDKN2A/B	
T233	++[EGFR, 2q34], +[1q21.2-24.2, Chr7, Chr19, Chr20], - [9p24.3 -13.3, Chr10]		
T238	+[Chr7, Chr19, Chr20] -[6q, Chr10, Chr13], --CDKN2A/B		
T239	+[Chr7, Chr19], -[1p36.32-p34.3, Chr10], --CDKN2A/B		
T251	++[EGFR, 2q34], +[1q21.2-24.2, Chr7, Chr19, Chr20], - [9p24.3 -13.3, Chr10]		
T281	-CDKN2A/B; low tumor content	+Chr7, - (partial Chr1, 3p, Chr6, Chr9, Chr10, Chr13, Chr14, Chr16, partial Chr17, -CDKN2A/B)	
T304	++[EGFR, CDK4, MDM2], +[Chr1, Chr7, Chr19, Chr20], -(partial Chr1, Chr10, partial Chr12)	0 (Chr19)	
T331	++EGFR, +[Chr1, Chr7, Chr20],- [9p, Chr10, Chr22], --CDKN2A		
T341	++[PDGFRA, CDK6], - [3q25.3-q26.2, Chr10], --CDKN2A/B		
T347	++EGFR, +[Chr7, 19p, Chr20], - [1p36.3-p34.3, 1q32.3-q44, Chr10, 13q12.11-31.1, 14q11.2-q21.3], --CDKN2A/B		
T356	low tumor content	+Chr7, -(partial Chr1, partial Chr2, partial Chr3, Chr4, Chr6, Chr10, Chr14, Ch17, Chr18)	
T361	++EGFR, + [Chr7, 19p, 20p13-p11.1, 20q13.1-q13.3], - [3q13.33-q21.1, 6p25.3-p21.32, 10q23.2-q26.3], --CDKN2A/B		
T367	++EGFR, +[Chr7, Chr19], - Chr10	++MDM4	
T384	++EGFR, low tumor content	Not assessed	Not assessed
T386	+ Chr7, - [1p36.3-p34.3, Chr10, 11q12.3-q13.2, 11q24.1-q13.2, 14q11.2-q24.1, 17q11.2-q21.2, 18p11.32-p11.22, Chr22], --CDKN2A/B		
T394	complex genome ++[PDGFRA,KIT, KDR, MET], --CDKN2A/B		
T407	complex genome ++[PDGFRA,KIT, KDR, MET], --CDKN2A/B		
T434	++[EGFR, CDK4, MDM2], +[7p,7q36.1-q36.2, Chr20], -[1p36.23-p36.21, 6q26-q27, Chr10, 14q13-qter]		
T470	++EGFR, +[Chr7, 19p, Chr20], - [1p36.3-p34.3, 1q32.3-q44, Chr10, 13q12.11-31.1, 14q11.2-q21.3], --CDKN2A/B	-1p31.1-p11.2	
T476	++[EGFR, MDM4] +[1p35.2-p35.1, 1q32.1, 3q26.1-q29, Chr7], - Chr10, -- CDKN2A/B		
T591	++EGFR, +Chr7, - [8q, partial Chr9, Chr10, 11q, Chr13, Chr15]	Not assessed	
T756	complex genome	Not assessed	
T772	++EGFR, +Chr7, - Chr10, --CDKN2A/B	Not assessed	
T784	+[Chr7, partial Chr3] - [partial Chr10, partial Chr13] --CDKN2A/B, additional partial chromosomal losses	Not assessed	
T797	++[PDGFRA, MDM2, CDK4], +Chr7, - [Chr4, Chr10, Chr11, partial Chr12, Chr13]	Not assessed	
T831/T832	++EGFR, +Chr7, - Chr10, --CDKN2A/B, additional partial chromosomal losses	Not assessed	

Supplementray Table 4 List of genetic variants private to patient tumors and respective PDOXs.

Private events	Compared to	Chromosome	Region	Reference	Allele	Read count	Read coverage	Frequency	Amino acid change	Coding region change	hg19_Genes	dbSNP	AC gnomad	AF gnomad
T101_Patient	T101_PDOX	3	12626516	G	A	56	250	22.4		NM_002880.3:c.1669-36C>T	RAF1	3729931	88513	0.35
T158_Patient	T158_PDOX	5	1293879	C	A	8	32	25		NM_198253.2:c.1122G>T	TERT			
T158_Patient	T158_PDOX	9	139414012	T	G	31	59	54.1	NP_060087.3:p.Thr250Pro	NM_017617.4:c.748A>C	NOTCH1			
T158_Patient	T158_PDOX	9	139414015	A	C	28	57	49.12	NP_060087.3:p.Phe249Val	NM_017617.4:c.745T>G	NOTCH1			
T158_Patient	T158_PDOX	10	104268859..104268860	GC	A	49	137	37.14		NM_016169.3:c.183-67_183-66delinsA	SUFU			
T158_Patient	T158_PDOX	10	104386934	T	C	50	113	44.25		NM_016169.3:c.1299T>C	SUFU	17114803	40291	0.16
T158_Patient	T158_PDOX	10	104387019	T	C	45	114	39.47		NM_016169.3:c.1365+19T>C	SUFU	12414407	165628	0.66
T158_Patient	T158_PDOX	10	123239112	G	A	61	180	33.89		NM_022970.3:c.*259C>T	FGFR2	1047057	85941	0.55
T158_Patient	T158_PDOX	10	123243197	G	A	45	106	42.45		NM_022970.3:c.2304+15C>T	FGFR2	2278202	138507	0.56
T158_Patient	T158_PDOX	10	123277507	G	A	51	171	29.82		NM_022970.3:c.1087+689C>T	FGFR2	117480075		
T158_Patient	T158_PDOX	10	123298158	T	C	41	159	25.79		NM_022970.3:c.696A>G	FGFR2	1047100	196571	0.78
T158_PDOX	T158_Patient	2	29451799	T	C	4	10	40		NM_004304.4:c.2766A>G	ALK	778543123	7339	0.04
T158_PDOX	T158_Patient	2	29451802	G	C	5	10	50	NP_004295.2:p.Phe921Leu	NM_004304.4:c.2763C>G	ALK	201042802	4651	0.03
T158_PDOX	T158_Patient	7	142640111	C	T	17	45	37.78	NP_000411.1:p.Ala598Thr	NM_000420.2:c.1792G>A	KEL	149066842	32	1.27E-04
T185_Patient	T185_PDOX	10	131565064	A	G	20	71	28.17	NP_002403.2:p.Ile174Val	NM_002412.4:c.520A>G	MGMT	2308321	22954	0.09
T186_Patient	T186_PDOX	6	33290602	G	C	6	25	24		NM_001141969.1:c.-53+37C>G	DAXX	1331292456	3448	0.02
T192_Patient	T192_PDOX	10	43622217	T	C	37	149	24.83		NM_020975.4:c.3187+47T>C	RET	2075912	185804	0.79
T233_Patient	T233_PDOX	10	43595968	A	G	28	110	25.45		NM_020975.4:c.135A>G	RET	1800858	1.83E+05	0.73
T233_Patient	T233_PDOX	10	43613843	G	T	23	103	22.33		NM_020975.4:c.2307G>T	RET	1800861	185430	0.74
T233_Patient	T233_PDOX	10	123239112	G	A	35	111	31.53		NM_022970.3:c.*259C>T	FGFR2	1047057	85941	0.55
T233_PDOX	T233_Patient	7	100676512	T	G	79	322	24.53	NP_001035194.1:p.Ser605Arg	NM_001040105.1:c.1815T>G	MUC17			
T233_PDOX	T233_Patient	7	148516122	T	C	47	109	43.12		NM_004456.4:c.999+566A>G	EZH2			
T238_Patient	T238_PDOX	6	87966524	T	C	33	94	35.11		NM_015021.1:c.3177T>C	ZNF292	143504993	998	4.02E-03
T238_Patient	T238_PDOX	6	114265587	T	C	48	134	35.82		NM_001527.3:c.1092-13A>G	HDAC2	13204445	57597	0.24
T238_Patient	T238_PDOX	9	2170607	G	A	14	47	29.79		NM_003070.4:c.4253+135G>A	SMARCA2	117087869		
T238_Patient	T238_PDOX	9	5081780	G	A	54	141	38.3		NM_001322194.1:c.2490G>A	JAK2, AL161450.1	2230724	132377	0.53
T238_Patient	T238_PDOX	10	70332580	A	G	51	141	36.17	NP_085128.2:p.Asp162Gly	NM_030625.2:c.485A>G	TET1	10823229	82847	0.33
T238_Patient	T238_PDOX	10	89653686	A	G	12	25	48		NM_001304718.1:c.-626-96A>G	PTEN	1903858		
T238_Patient	T238_PDOX	10	104268877	G	C	106	220	48.18		NM_016169.3:c.183-49G>C	SUFU	2281879	85332	0.34
T238_Patient	T238_PDOX	10	123239112	G	A	99	308	32.25		NM_022970.3:c.*259C>T	FGFR2	1047057	85941	0.55
T238_Patient	T238_PDOX	10	123243197	G	A	31	104	29.81		NM_022970.3:c.2304+15C>T	FGFR2	2278202	138507	0.56
T238_Patient	T238_PDOX	10	123298158	T	C	55	157	35.03		NM_022970.3:c.696A>G	FGFR2	1047100	196571	0.78
T238_Patient	T238_PDOX	13	28589495	C	G	46	117	39.32		NM_004119.2:c.2654-102G>C	FLT3	9579142		
T238_Patient	T238_PDOX	13	32890572	G	A	68	170	40		NM_000059.3:c.-26G>A	BRCA2	1799943	61394	0.25
T238_Patient	T238_PDOX	13	32911888	A	G	64	170	37.65		NM_000059.3:c.3396A>G	BRCA2	1801406	73766	0.29
T238_Patient	T238_PDOX	13	32929232	A	G	40	113	35.71		NM_000059.3:c.7242A>G	BRCA2	1799955	56617	0.23
T238_Patient	T238_PDOX	13	32936646	T	C	43	103	41.75		NM_000059.3:c.7806-14T>C	BRCA2	9534262	130837	0.52
T238_Patient	T238_PDOX	13	32953388	T	C	30	71	42.25		NM_000059.3:c.8755-66T>C	BRCA2	4942486		
T238_Patient	T238_PDOX	17	29555971	A	G	28	74	37.84		NM_001042492.2:c.2410-72A>G	NF1	777315699		
T251_Patient	T251_PDOX	X	39922282..39922285	GAAA	-	20	79	25.32	NP_001116857.1:p.Leu1296fs	NM_001123385.1:c.3887_3890del				
T331_PDOX	T331_Patient	7	100694854	G	A	9	39	23.08		NM_001040105.1:c.12875-40G>A	MUC17	67328257	74616	3.00E-01
T331_PDOX	T331_Patient	9	5050706	C	T	34	121	28.1		NM_001322194.1:c.489C>T	JAK2	2230722	80745	0.32
T331_PDOX	T331_Patient	9	5081780	G	A	33	97	34.02		NM_001322194.1:c.2490G>A	JAK2	2230724	132377	0.53
T331_PDOX	T331_Patient	10	43606687	A	G	58	185	31.35		NM_020975.4:c.1296A>G	RET	1800860	174813	0.7
T331_PDOX	T331_Patient	22	21344884	C	T	14	63	22.22		NM_006767.3:c.791+70C>T	LZTR1	2073989		
T331_PDOX	T331_Patient	22	24167513	G	A	52	152	34.21		NM_001317946.1:c.924G>A	SMARCB1	2229354	28247	0.11
T341_Patient	T341_PDOX	7	151935962	A	C	9	30	30		NM_170606.2:c.2533-51T>G	KMT2C	113178923		
T341_PDOX	T341_Patient	4	55138664	G	C	357	464	76.89	NP_001334579.1:p.Trp460Cys	NM_001347830.1:c.1380G>C	PDGFRA			
T407_Patient	T407_PDOX	14	95591070	G	A	21	57	36.84		NM_030621.4:c.904-65C>T	DICER1	67737119		
T407_Patient	T407_PDOX	14	105239146	C	G	12	35	34.29		NM_005163.2:c.1172+69G>C	AKT1	3803304		
T407_Patient	T407_PDOX	16	2105400	C	T	102	203	50.25		NM_000548.4:c.482-3C>T	TSC2	1800720	21836	0.09
T407_Patient	T407_PDOX	16	2115481	C	T	84	157	53.5		NM_000548.4:c.1600-39C>T	TSC2	45477195	18357	0.07
T434_PDOX	T434_Patient	12	18762483	G	C	17	65	26.15	NP_001275701.1:p.Asp1368His	NM_001288772.1:c.4102G>C	PIK3C2G			
T470_PDOX	T470_Patient	8	90948273	C	T	7	28	25		NM_001024688.2:c.1989-433G>A	NBN	2735384		
T476_Patient	T476_PDOX	9	2116037	G	A	12	48	25		NM_003070.4:c.3672G>A	SMARCA2	6601	30639	0.12

Supplementary Table 5 **Glioma specific mutations in patient tumors and preclinical models.**

Mutations were established form targeted DNA sequencing and are displayed for a selection of glioma-relevant driver genes.
* P8 and T16* patient tumors sequenced correspond to clones 2 from aCGH

Gene	PDOX model	P3	P8 *	P13	T16 *	T101	T158	T185	T186	T188	T192	T233	T238	T251	T331	T341	T347	T361	T394	T407	T434	T470	T476
IDH1	Patient								R132H, NM_005896										R132H, NM_005896	R132H, NM_005896			
	PDOX								R132H, NM_005896										R132H, NM_005896	R132H, NM_005896			
	Cell line																		R132H, NM_005896	R132H, NM_005896			
IDH2	Patient																						
	PDOX			W244R, NM_002168																			
	Cell line			W244R, NM_002168																			
ATRX	Patient		S1348T, NM_000489						Glu1511*, NM_000489										D1212del E886fs , NM_000489	D1212del E886fs , NM_000489			
	PDOX		S1348T, NM_000489						Glu1511*, NM_000489										D1212del E886fs , NM_000489	D1212del E886fs , NM_000489			
	Cell line																		D1212del E886fs , NM_000489	D1212del E886fs , NM_000489			
TP53	Patient																		E204fs, NM_001126115	E204fs, NM_001126115			
	PDOX	C176Y, NM_001126112.2																	E204fs, NM_001126115	E204fs, NM_001126115			
	Cell line	C176Y, NM_001126112.2																	E204fs, NM_001126115	E204fs, NM_001126115			
EGFR	Patient		A289T, NM_005228, ex10-11del		ex2-7del			G598V, NM_005228		ex-6-7del, ex25-26del	complex rearrangements (ex1-6), ex14-15	vIII, complex rearrangements		vIII, complex rearrangements	R108K, NM_005228		ex2-15del	R108K, NM_005228			F254I, NM_005228	ex2-15del	
	PDOX		A289T, NM_005228		ex25-27del			G598V, NM_005228		ex-6-7del, ex25-26del	complex rearrangements (ex1-6), ex14-16	vIII, complex rearrangements		vIII, complex rearrangements	R108K, NM_005228		ex2-15del	R108K, NM_005228			F254I, NM_005228	ex2-15del	G598V,NM_005228, complex rearrangements ex5-28
	Cell line			ex23-25del															F254I, NM_005228				
MDM4	Patient									K374Q, NM_002393			T222R, NM_002393		K374Q, NM_002393								
	PDOX									K374Q, NM_002393			T222R, NM_002393		K374Q, NM_002393								
	Cell line																						
MET	Patient		A954S, NM_001127500																				
	PDOX		A954S, NM_001127500																				
	Cell line																						
NF1	Patient							E244G, NM_001042492															
	PDOX							E244G, NM_001042492															
	Cell line																						
PIK3CA	Patient															G118N, NM_006218							
	PDOX															G118N, NM_006218							
	Cell line			A990V, NM_006218																			
PIK3R1	Patient						G376R, NM_181523																
	PDOX																						
	Cell line																						
PTCH1	Patient									V866I, NM_001083602			Y1250C, NM_001083602										
	PDOX									V866I, NM_001083602			Y1250C, NM_001083602										
	Cell line																						
PTEN	Patient		T319fs, NM_000314														C304fs, NM_000314				C136Y, NM_000314	C304fs, NM_000314	
	PDOX	D236fs, NM_000314	T319fs, NM_000314	D107V, NM_000314									R335*, NM_000314				C304fs, NM_000314				C136Y, NM_000314	C304fs, NM_000314	
	Cell line	L220fs, NM_000314		D107V, NM_000314									R335*, NM_000314										
RET	Patient								L56M, NM_020975														
	PDOX								L56M, NM_020975														
	Cell line																						
STAG2	Patient							T1151A, NM_001042749															
	PDOX							T1151A, NM_001042749															
	Cell line																						

Supplementary Table 6 **Classification of Glioma patient tumors and preclinical models based on the DNA methylation profiles.**

Detailed overview of the comparison of patient-, PDOX- and GSC line-related methylation-based molecular subgrouping and MGMT promoter methylation status (methylated vs. unmethylated).

Molecular diagnosis and MGMT promoter status are based on the analyses from the Heidelberg neuropathology tool https://www.molecularneuropathology.org/mnp, methylation class and methylation cluster are based on deSouza et al., 2019.

PDOX model	Molecular diagnosis Patient	Molecular diagnosis PDOX	Molecular diagnosis cell line	MGMT methylation Patient	MGMT methylation PDOX	MGMT methylation cell line	Methylation cluster patient	Methylation cluster PDOX	Methylation cluster cell line	Methylation class patient	Methylation class PDOX	Methylation class cell line
P8 (clone 2*)	GBM, IDHwt, RTK I	GBM, IDHwt, RTK I/II		unmethylated	methylated		LGm5	LGm4/5		Mesenchymal-like	Classic-like	
P3	NA	GBM, IDHwt, RTK II/I	No match	methylated	methylated	methylated	NA	LGm4	LGm4	NA	Classic-like	Classic-like
P13	NA	GBM, IDHwt, RTK II	No match	methylated	methylated	methylated	NA	LGm4	LGm4	NA	Classic-like	Classic-like
T16 (clone 2*)	GBM, IDHwt, mesenchymal	GBM, IDHwt, RTK II	No match	unmethylated	unmethylated	unmethylated	LGm5	LGm4	LGm4	Mesenchymal-like	Classic-like	Classic-like
T101	NA	GBM, IDHwt, RTK II		methylated	methylated		NA	LGm4		NA	Classic-like	
T158	GBM, IDHwt, mesenchymal	GBM, IDHwt, RTK II	NA	unmethylated	unmethylated	NA	LGm5	LGm4	NA	Mesenchymal-like	Classic-like	NA
T185	GBM, IDHwt, RTK II	GBM, IDHwt, RTK II		methylated	methylated		LGm4	LGm4		Classic-like	Classic-like	
T186	Glioma, IDHm, high grade astrocytoma	Glioma, IDHm, high grade astrocytoma		methylated	methylated		LGm1	LGm1		K1: G-CIMP-low	K1: G-CIMP-low	
T188	GBM, IDHwt, RTK II	GBM, IDHwt, RTK II		unmethylated	unmethylated		LGm4/5	LGm4/5		Classic-like	Classic-like	
T192	GBM, IDHwt, mesenchymal	GBM, IDHwt, RTK II		unmethylated	unmethylated		LGm5	LGm5		Mesenchymal-like	Mesenchymal-like	
T226	NA, low tumor content	GBM, IDHwt, RTK I		NA, low tumor content	methylated		LGm6, low tumor content	LGm4		LGm6, low tumor content	Classic-like	
T233	GBM, IDHwt, mesenchymal	GBM, IDHwt, RTK II		unmethylated	unmethylated		LGm5	LGm5		Mesenchymal-like	Mesenchymal-like	
T238	GBM, IDHwt, inflamatory tissue	GBM, IDHwt, RTK II		methylated	methylated		LGm5	LGm4/5		Mesenchymal-like	Classic-like	
T239	GBM, IDHwt, mesenchymal	GBM, IDHwt, RTK II		methylated	methylated		LGm4	LGm4		Classic-like	Classic-like	
T251	GBM, IDHwt, RTK II/ mesenchymal	GBM, IDHwt, RTK II		unmethylated	unmethylated		LGm5	LGm5		Mesenchymal-like	Mesenchymal-like	
T281	GBM, IDHwt, mesenchymal/ RTK II	GBM, IDHwt, RTK I/II		methylated	methylated		LGm4/5	LGm4		Classic-like	Classic-like	
T304	GBM, IDHwt, RTK II/I	GBM, IDHwt, RTK I/II		unmethylated	unmethylated		LGm4	LGm4		Classic-like	Classic-like	
T331	GBM, IDHwt, mesenchymal	GBM, IDHwt, RTK II		methylated	methylated		LGm5	LGm5		Mesenchymal-like	Mesenchymal-like	
T341	GBM, IDHwt, RTK I	GBM, IDHwt, RTK I		methylated	methylated		LGm4/5	LGm4/5		Classic-like	Classic-like	
T347	GBM, IDHwt, RTK II	GBM, IDHwt, RTK II		unmethylated	unmethylated		LGm4	LGm4		Classic-like	Classic-like	
T356	GBM, IDHwt, mesenchymal	GBM, IDHwt, RTK II/I		methylated (low tumor content)	unmethylated		LGm5	LGm5		Mesenchymal-like	Mesenchymal-like	
T361	GBM, IDHwt, RTK II	GBM, IDHwt, RTK II		methylated	methylated		LGm4	LGm4		Classic-like	Classic-like	
T367	GBM, IDHwt, RTK II	GBM, IDHwt, RTK I/II		methylated	methylated		LGm4/5	LGm4/5		Classic-like	Classic-like	
T386	GBM, IDHwt, RTK I	GBM, IDHwt, RTK I		unmethylated	unmethylated		LGm4	LGm4		Classic-like	Classic-like	
T394	Glioma, IDHm, high grade astrocytoma	Glioma, IDHm, high grade astrocytoma	Glioma, IDHm, high grade astrocytoma	methylated	methylated	methylated	LGm1	LGm1	LGm1	K1: G-CIMP-low	K1: G-CIMP-low	K1: G-CIMP-intermediate
T407	Glioma, IDHm, high grade astrocytoma	Glioma, IDHm, high grade astrocytoma	Glioma, IDHm, high grade astrocytoma	methylated	methylated	methylated	LGm1	LGm1	LGm1	K1: G-CIMP-low	K1: G-CIMP-low	K1: G-CIMP-intermediate
T434	GBM, IDHwt, RTK II	GBM, IDHwt, RTK II		unmethylated	unmethylated		LGm4	LGm4		Classic-like	Classic-like	
T470	GBM, IDHwt, RTK II	GBM, IDHwt, RTK II		unmethylated	unmethylated		LGm4	LGm4		Classic-like	Classic-like	
T476	GBM, IDHwt, RTK II/I	GBM, IDHwt, RTK I/II		unmethylated	unmethylated		LGm5	LGm5		Mesenchymal-like	Mesenchymal-like	

*Clones not propagated in PDOXs

Supplementary Table 7 **Verhaak glioma subtypes classification.**

Classification was performed for patient tumors and respective PDOX models as well as cell lines in vitro and xenografts derived thereof.
Classification was based on initial Verhaak 2010 signatures (Verhaak et al., 2010) and the tumor intrinsic 2017 signatures (Wang et al., 2017).

Classifier	Verhaak et al. 2010		Wang et al. 2017	
PDOXs	Patient	PDOX	Patient	PDOX
T16	Neural	Classical	Classical	Classical
T101	Mesenchymal	Classical	Classical	Classical
T185	Mesenchymal	Classical	Classical	Classical
P3	Mesenchymal	Classical	Classical	Classical
P8	Neural	Proneural	Classical	Classical
P13	Neural/Mesenchymal	Classical	Classical	Classical
Cell lines	Cell line	Xenograft	Cell line	Xenograft
NCH421k	Proneural	Proneural	Proneural	Proneural
NCH644	Proneural	Proneural	Proneural	Proneural
NCH601	NA	NA	Mesenchymal/Classical	Classical
U87	Mesenchymal	Mesenchymal	Mesenchymal	Mesenchymal
U251	Mesenchymal	Mesenchymal	Mesenchymal	Mesenchymal

Supplementary Table 8

Quantification of AUC in PDOX-derived organoids upon exposure to EGFR and CDK4/6 inhibitors.

Mean AUC values +/- SEM are shown for each PDOX. Experiments were performed twice with 3 technical replicates each.
SEM represents variation between biological replicates.
Table relates to Fig. 6f and Supplementary Fig. 6g.

**PDOX model**	**Erlotinib**		**Gefitinib**		**Abemaciclib**		**Palbociclib**		**AG-490**		**Daphnetin**		**AZD3759**	
	AUC	SEM	AUC	SEM	AUC	SEM	AUC	SEM	AUC	SEM	AUC	SEM	AUC	SEM
**P13**	422.75	15.9453	369.95	11.1369	285.7	10.0409	390.6	3.67696	407.1	12.8693	366.5	4.10122	408.45	9.93485
**P3**	380.9	5.79828	322.9	4.24264	281.2	6.36396	319.5	31.537	356.85	23.2992	354	17.6777	347.4	16.5463
**P8**	218.7	11.5966	261.25	0.95459	388.25	2.22739	386.1	13.0108	382.9	7.00036	395.85	23.0163	290	14.425
**T101**	415.6	19.6576	330.45	2.79307	332.75	7.81353	357.1	9.33381	408.2	16.9706	380.55	3.0052	359.6	12.3037
**T16**	313.4	5.72756	291.1	1.90919	332.65	9.72272	350.85	11.9147	336.8	19.3747	357.45	4.77297	326.45	14.1068
**T185**	263.05	7.81353	326.35	3.85373	268.75	0.81317	352.1	13.0815	348.1	11.8087	339.25	15.6624	269.15	16.7231
**T188**	321.4	5.9397	351.45	4.56084	359.8	8.98026	417.15	4.50462	338.45	9.79343	323.2	6.22254	370.7	3.88909
**T192**	294.15	10.8541	263.7	7.9196	204.85	8.16708	320.3	32.739	375.3	9.05097	353.05	13.3997	307	11.8087
**T251**	252.55	2.79307	255.9	11.0309	240.1	23.9709	334.25	30.2995	330	2.12132	341.9	29.5571	289.45	2.22739
**T331**	263.7	2.89914	229.45	21.2486	292.55	13.8239	366.35	22.7335	299.1	1.83848	325.9	6.57609	249.1	7.14178
**T341**	399.75	4.52195	361.4	5.3033	245.8	11.65	273.65	19.8343	383.45	22.8042	393.75	28.461	364.9	24.3245
**T347**	384.05	5.33866	380.5	0.91924	326.35	5.97505	413.7	7.14178	361.9	0.28284	389.8	1.83848	359.45	0.24749
**T361**	309.45	1.52028	292.5	10.3238	335.75	17.2888	353.05	9.72272	375.45	7.88424	356.75	2.79307	311.7	23.9002
**T434**	391.45	2.72236	335.85	1.0253	206	18.6676	255.7	10.5359	384	1.48492	423.65	7.88424	381.5	0.98995
**T470**	382.6	19.47	307.5	13.93	340.9	16.84	323.3	17.2	359.3	8.594	326	13.2	314.7	17.88
**T476**	272.5	10.13	307.1	9.305	314	11.55	327.1	14.17	373.4	14.36	367.1	17.02	302.2	15.16
